# The Role of Noncovalent Interactions in the Infrared Spectra of Lignin Model Compounds: A DFT Study

**DOI:** 10.3390/molecules30244694

**Published:** 2025-12-08

**Authors:** Febdian Rusydi, Lusia Silfia Pulo Boli, Indri Badria Adilina, Wahyu Tri Cahyanto, Stewart F. Parker, Ferensa Oemry

**Affiliations:** 1 Research Center for Quantum Engineering Design, Faculty of Science and Technology, Universitas Airlangga, Jl. Mulyorejo, Surabaya 60115, East Java, Indonesia; rusydi@fst.unair.ac.id; 2 Physics Study Program, Universitas Jenderal Soedirman, Jl. Dr. Soeaparno 61, Purwokerto 53123, Central Java, Indonesia; lusia.silfia@unsoed.ac.id (L.S.P.B.); wahyu.cahyanto@unsoed.ac.id (W.T.C.); 3 Research Center for Catalysis, National Research and Innovation Agency, KST BJ Habibie, Tangerang Selatan 15314, Banten, Indonesia; indri030@brin.go.id; 4 ISIS Neutron and Muon Source, STFC Rutherford Appleton Laboratory, Chilton, Didcot OX11 0QX, Oxfordshire, UK; 5 Research Center for Quantum Physics, National Research and Innovation Agency, KST BJ Habibie, Tangerang Selatan 15314, Banten, Indonesia

**Keywords:** lignin, monomer, dimer, noncovalent interaction, density functional theory

## Abstract

Noncovalent interactions are key to the stability of lignin dimers, the primary components of bio-oil. However, their specific influence on the infrared spectra remains poorly understood. Using dispersion-corrected density-functional theory (APFD/6-311++G(d,p)), we conducted a comparative analysis of the structures and infrared spectra of four lignin derivatives—benzene, phenol, anisole, and guaiacol—as monomers and dimers. Our study reveals that distinct vibrational shifts and newly emerging peaks observed in the calculated infrared spectra of the dimers can be attributed to the formation of σ···π and π···π stacking, hydrogen bonding, attractive van der Waals, and steric repulsion interactions. We extended the study to 3,3′-dimethoxy-1,1′-biphenyl-2,2′-diol, a guaiacyl moiety with a C–C linkage. The results indicate that the structure in which both guaiacol units adopt an anti–syn conformation is more stable—minimizing steric repulsion between hydroxyl groups—than configurations in which one guaiacol unit adopts either anti–anti or gauche–anti conformation. These differences are clearly reflected in their distinctive infrared spectral signatures.

## 1. Introduction

Agricultural residues are increasingly investigated as feedstocks for renewable energy production, driven by the need to reduce dependence on fossil fuels, the primary sources of air pollution and greenhouse gas emissions [1,2,3]. These residues, classified as lignocellulosic biomass, contain high levels of cellulose, hemicellulose, and lignin, making them promising candidates for biofuels and other energy-related applications [2,3,4]. Depending on the source, lignocellulose typically consists of 40–50% cellulose, 15–30% hemicellulose, and 20–30% lignin [5]. Lignin, the only biopolymer rich in aromatic structures, is particularly attractive for the production of polymers [4,6], bioplastics [4], batteries [6,7], drug deliveries [6], biofuels [2,3] and biomedical applications [4]. Lignin valorization for biofuel production generally involves three steps: (i) pretreatment of lignocellulosic biomass to isolate lignin [2]; (ii) lignin depolymerization [5]; (iii) bio-oil upgrading using heterogeneous catalysts [8,9]. The pretreatment methods in the first step depolymerize the lignocellulosic biomass into three main components (cellulose, hemicellulose, and lignin). Structurally, lignin originates from three monolignols with varying degrees of methoxylation—p-coumaryl, coniferyl and sinapyl alcohols—yielding the p-hydroxyphenyl (H), guaiacyl (G) and syringyl (S) units, respectively [6,10].

In the second step, lignin is typically decomposed by pyrolysis into monomers, dimers, or oligomers. Pyrolysis is a thermochemical process carried out in the absence of oxygen at 300–1000 °C [2,11]. Depending on operating conditions, it is classified as slow, fast, flash, or hydro-pyrolysis [2]. Among these, fast pyrolysis is most widely applied, producing fuel-range compounds and value-added chemicals at 450–650 °C, with typical yields of 60–75% liquid bio-oil, 10–20% gases (CO_2_, CO, CH_4_, H_2_) and 15–25% solid biochar [3]. The resulting bio-oil is a complex mixture of over 100 organic compounds, primarily aromatic moieties with diverse functional groups. Kumar and coworkers [3] categorized these oxygenated compounds into seven groups: aromatic hydrocarbons, aldehydes, ketones, alcohols, phenolics, ethers, and furans. Phenolic products include phenol, anisole, guaiacol, m-cresol, p-cresol, and catechol [3,8], while aromatic hydrocarbons are limited to benzene, toluene, o-xylene, 2-methylnaphthalene, and 1,3-dimethylnaphthalene [3,8,12].

In lignin model compounds [6,8,13,14], monomers are represented by molecular structures with one aromatic ring while dimers, trimers, tetramers and beyond are characterised by two, three, four and more aromatic species coupled and connected by various ether (β–O–4, α–O–4, 4–O–5) and C–C (β–5, 5–5, β–1, and β–β) linkages [3,6,10]. All of them are oligomers, typically ranging from dimers (two units of aromatic rings) to about 10–20 units. Previous studies on fast pyrolysis of corn stover [11,15] show that most oligomers observed in lignin-derived bio-oil are mainly monomers, dimers, trimers, tetramers, and pentamers with an uneven distribution. Monomers and dimers are found to be the most abundant lignin components in the bio-oil. However, the reason behind these high yields of monomers and dimers from lignin depolymerization are rarely explained due to a limited understanding of the mechanisms governing their polymerization. Therefore, unlocking a fundamental understanding of the polymerization mechanisms of lignin oligomers that lead to the formation of the complex lignin polymer is necessary, as it would pave the way to reverse engineering of the depolymerization processes.

Several attempts have been made to investigate the polymerization process of lignin from its smallest unit monomers. LigninGraphs [16], a metropolis Monte Carlo-based simulation, has shown a potent practicability to generate complex lignin structures that closely match experimental data by coupling the monomers sequentially with intramolecular (ether or C–C) linkages. Another study unravels a fundamental insight into lignin polymerization processes where DFT and experimental data indicate that intramolecular π···π stacking in β–O–4-rich segments brings reactive sites into proximity and thereby facilitates the observed selective intramolecular condensation [17]. The role of π···π stacking in lignin polymerization has been elaborated previously by DFT [18] which concluded that hydrogen bonding and π···π stacking between two monolignols initiate and lead the formation of the β–O–4 and C–C linkages. Here, π···π stacking can be seen as an initial, noncovalent step where two or more aromatic moieties in bio-oil come close and orient in a favorable geometry. In addition, π···π stacking is an indicator for face-to-face attractive forces between aromatic hydrocarbons such as polycyclic aromatic hydrocarbons (PAHs) [19], one of the coke precursors which deactivates catalysts during hydrodeoxygenation (HDO) process [20].

Previous studies on non-bonding interactions (hydrogen bonding, π···π stacking, van der Waals, etc.) in benzene, phenol, anisole, and guaiacol dimers have combined spectroscopic and computational approaches [21,22,23,24,25,26]. These studies primarily investigate peak shifts and the appearance of new peaks in several isomers containing one to four monomers, assigning the corresponding infrared (IR) bands to specific vibrational modes. Computational methods were primarily used to interpret microwave, infrared, Raman, and UV spectra. Although IR peaks indicative of hydrogen bonding are frequently noted, they have rarely been examined in detail because earlier computational tools were limited in their ability to explicitly account for such long-range interactions [27]. This might explain why the role of noncovalent interactions in determining dimer configurations in lignin models has rarely been addressed. To fill this gap, we performed density functional theory (DFT) calculations on dimers of four lignin model monomers (benzene, phenol, anisole, and guaiacol). For stable isomer searches for dimers, several possible conformers are considered: parallel face-centered, parallel offset, and perpendicular T-shaped. Noncovalent interaction (NCI) [28,29,30,31] analysis was employed to quantify interaction strengths, locate noncovalent contacts, and interpret IR spectral shifts between monomers and dimers. Finally, we investigated a guaiacyl moiety with a C–C linkage (3,3′-dimethoxy-1,1′-biphenyl-2,2′-diol) [22] to assess how the orientation of functional groups affects the dihedral angle between the aromatic rings, as evidenced by distinctive IR peaks confirmed through NCI analysis. This provides a direct comparison of the IR spectral profiles of two guaiacol units in a dimer configuration with, and without, a C–C linkage.

## 2. Results and Discussion

### 2.1. Lignin Model Compounds

**Figure 1:**
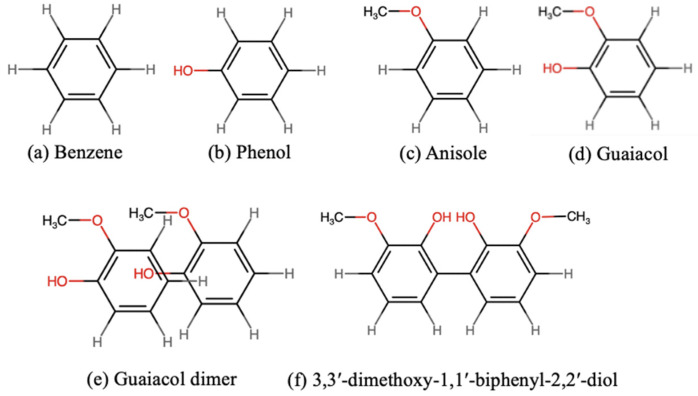
Structures of lignin model compounds: monomers (**a**–**d**), a guaiacol dimer (**e**) and a guaiacyl moiety (3,3′-dimethoxy-2,2′-biphenyl-diol) adopted from ref. [22] (**f**). Atoms in red indicate oxygen-containing functionalities.

We modeled lignin compounds using four monomers and their corresponding dimers without a C–C linkage. The monomers were benzene, phenol, anisole, and guaiacol (Figure 1a–d). The dimers were constructed based on four configurations reported in the previous literature [32,33,34]: parallel face-centered (ParF), parallel offset (ParO), perpendicular T-shaped (PerT) and perpendicular Y-shaped (PerY). For dimers with substituents, we also considered several distinct variations of each configuration. An example of a ParO guaiacol dimer is shown in Figure 1e, while other configurations are presented in Figure S1. The optimized structures were named according to their final configurations.

We used two guaiacol monomer to create a model lignin compound with a C–C linkage, as illustrated in Figure 1f. The model was adopted from reference [22]. Considering that conformational effects can affect molecular stability [35], we examined several conformations of guaiacol with a C–C linkage. Conformers were generated by rotating the hydroxyl and the methoxy groups on both aromatic rings. Rotational variations corresponding to cis (c), gauche (g), anticlinal (a) and trans (t) conformations were examined, yielding seven distinct stable conformers (Table S1). Each conformer was named according to the conformations of the substituents on both rings. When two or more conformers shared the same name, they were distinguished by the dihedral angle between the two aromatic rings, for example, ct-ct-60.

### 2.2. Geometries and Thermochemical Analysis

**Figure 2:**
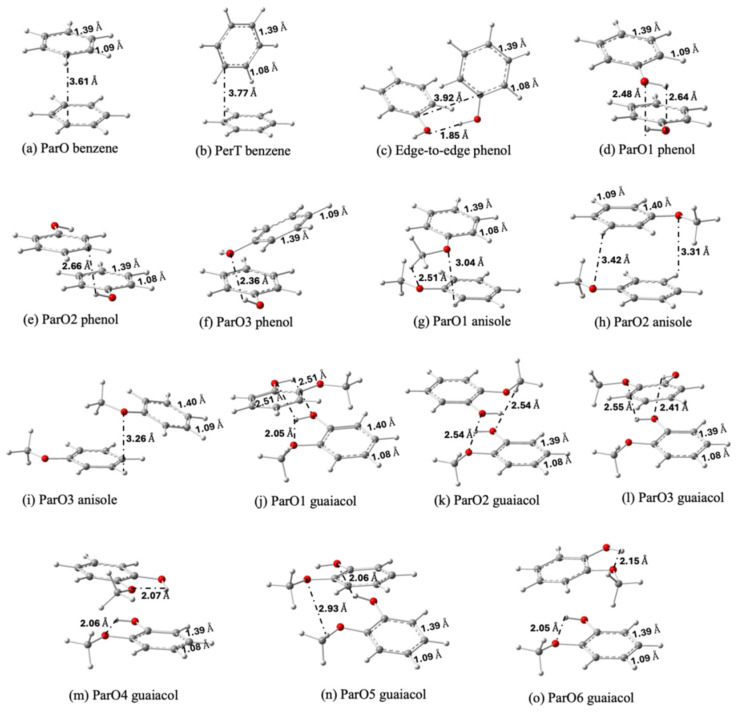
Optimized geometries of benzene (**a**,**b**), phenol (**c**–**f**), anisole (**g**–**i**) and guaiacol (**j**–**o**) dimers from the most to the least stable configurations. White, grey, and red atoms represent hydrogen, carbon, and oxygen atoms, respectively.

**Figure 3:**
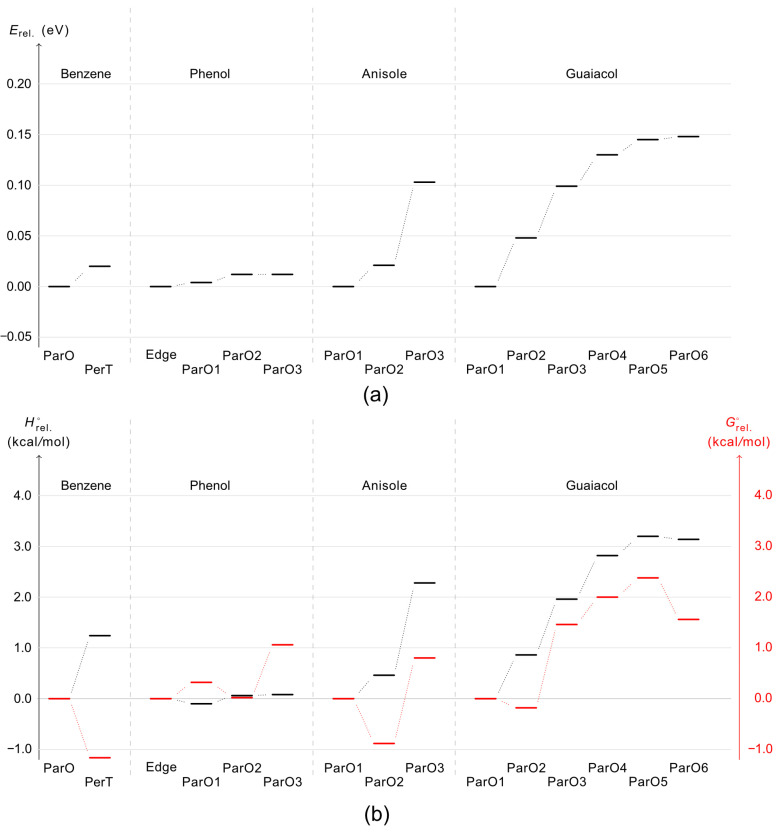
Stability order of each dimer configuration according to their relative electronic energy ($E_{rel}$) (**a**) and enthalpy change (Δ$H_{rel}^{{}^{\circ}}$) and Gibbs free energy change (Δ$G_{rel}^{{}^{\circ}}$) (**b**). ParF, ParO, PerT and Edge stand for parallel face-centered, parallel offset, perpendicular T-shaped, and edge-to-edge dimer configurations.

Figure 2 displays the stable dimer configurations of benzene, anisole, phenol, and guaiacol, identified through geometry optimization. The relative stability of these conformers is quantified in Figure 3a. Our calculations show a strong preference for the ParO configuration, which constitutes the global minimum for all systems except phenol. This prevalence indicates that the ParO configuration optimally facilitates stabilizing the interactions between the two aromatic rings. The exceptional stability of the edge-to-edge phenol dimer (Figure 2c), however, suggests a stronger inter-molecular interaction which outweighs the aromatic ring interaction available in its ParO forms.

The stability of the ParO configuration is attributed to attractive electrostatic interactions between regions of opposing partial charge. The electrostatic potential (ESP) maps in Figure S2 illustrates this interaction. For example, in the benzene monomer, the electron-rich region is located within the ring, while the electron-deficient region is distributed around the hydrogen atoms. In the ParO dimer configuration (Figure 2a), these complementary regions are optimally aligned, with the positive edge of one monomer facing the negative face of the other. This results in a stabilizing electrostatic interaction, a driving force also observed in the ParO1 anisole (Figure 2g) and guaiacol (Figure 2j) dimers. This interaction has also been proposed previously as the driving force that determines the geometry of interaction [36,37].

Since IR measurements are typically conducted at or near room temperature, it is reasonable to include thermochemical corrections to the relative electronic energy ($E_{rel}$ at 0 K) to evaluate whether the previously discussed stable conformers remain thermodynamically favoured at 298 K once thermal and entropic effects are considered. This can be verified by examining their enthalpy change and Gibbs free energy change (ΔH and ΔG) values. Figure 3b presents the stability of these conformers based on their relative enthalpy change (Δ$H_{rel}^{{}^{\circ}}$) and Gibbs free energy change (Δ$G_{rel}^{{}^{\circ}}$) values for dimer formation, where their relative enthalpy and Gibbs free energy provided in Figure S3. Overall, the conformers with the lowest Δ$H_{rel}^{{}^{\circ}}$ (and $H_{rel}^{{}^{\circ}}$) values exhibit a stability trend consistent with that derived from their lowest $E_{rel}$ values, except in the case of phenol, where the most stable configuration shifts from the Edge-to-edge to the ParO1 structure. However, the Δ$G_{rel}^{{}^{\circ}}$ values indicate that the most stable dimers at 0 K and 298 K may differ due to entropic contributions, with phenol being the only exception where the Edge-to-edge configuration remains the most stable at both temperatures. For benzene, anisole, and guaiacol, the most stable configurations shift to those that were second-most stable at 0 K when thermal corrections are applied. Specifically, the most stable conformers for benzene, anisole, and guaiacol at 298 K are the PerT, ParO2, and ParO2 configurations, respectively. To further confirm the reliability of the calculated Δ$H_{rel}^{{}^{\circ}}$ and Δ$G_{rel}^{{}^{\circ}}$ values, the transition of the benzene dimer from the ParO to the PerT configuration with increasing temperature will be examined.

A previous theoretical study employing on-the-fly ab initio molecular dynamics simulations [38] predicted that both parallel displaced (ParO) and T-shaped (PerT) benzene dimer structures are present at 10 K. At higher temperatures, the T-shaped configuration becomes more dominant due to its favorable entropic contribution to the free energy. Direct IR absorption spectroscopy measurements on the benzene dimer in the gas phase using jet-expansion [39] confirmed the existence of the T-shaped structure; however, the presence of the parallel-displaced configuration could not be definitively confirmed or ruled out. Our calculations show a similar trend, in which the PerT structure exhibits a lower Δ$G_{rel}^{{}^{\circ}}$ value (−1.2 kcal·mol^−1^) with respect to the ParO. This result indirectly suggests that the T-shaped structure is the predominant benzene dimer at 298 K. Given the consistency with previous theoretical and experimental findings, the calculated Δ$G_{rel}^{{}^{\circ}}$ values for phenol, anisole, and guaiacol are expected to reliably indicate the most stable configurations at 298 K.

### 2.3. Noncovalent Interaction (NCI) Analysis of Dimers

**Figure 4:**
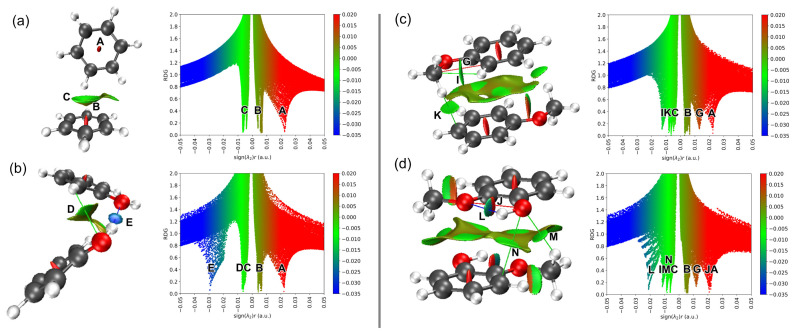
NCI plots of the reduced density gradient (RDG) iso-surfaces (left) and its associated 2D (s, *ρ*) diagram (right) for the most stable benzene (**a**), phenol (**b**), anisole (**c**) and guaiacol (**d**) dimers based on the lowest Δ$G_{rel}^{{}^{\circ}}$ values. The surfaces are colored on a blue-green-red scale according to values of sign (λ_2_)*ρ*, ranging from −0.05 to +0.05 au. Blue, green and red colors denote strong attractive interaction, weak interaction, and strong nonbonded overlap, respectively. The letters denote interaction type as listed in Table 1.

Lignin monomers can interact to form either new molecules (reactive channel) or molecular clusters (nonreactive channel). The former involves covalent interactions of aromatic molecules linked by ether and C–C bonds [6,10], while the latter, shown in Figure 2, where a covalent bond is neither formed nor broken, is termed noncovalent or van der Waals (vdW) interactions. The stable dimer configurations interact at distances around 2–4 Å with stabilization energies below 23 kcal/mol (1 eV), much lower than covalent bond energies (23–100 kcal/mol). Analyzing these non-covalent interactions is now feasible with functionals that include vdW interactions in density functional theory (DFT) calculations [40,41] and the implementation of the NCI method for molecules [29,31] and solids [28,30]. The NCI visualization method [28,31] can identify and visualize non-covalent interactions in three-dimensional space, distinguishing different interaction types by examining the reduced density gradient (RDG) and the sign of the Laplacian of the electron density. Figure 4 shows the NCI RDG plots of the most stable benzene, phenol, anisole, and guaiacol dimers at 298 K, while the NCI plots of their stable monomers and dimer counterparts at 0 K are provided in Figures S4 and S5 (see Supplementary Materials).

The NCI method, originally developed by Johnson et al. [29,30], provides a visual representation of weak intermolecular interactions directly in real space. The analysis is based on the reduced density gradient:

(1) $$s\left(r\right)=\frac{1}{2{\left(3\pi^{2}\right)}^{1/3}}\frac{\left|\nabla \rho (r)\right|}{\rho {\left(r\right)}^{4/3}}$$

where ρ is the electron density. Regions of low ρ and small s correspond to noncovalent interactions such as hydrogen bonds, π–π stacking and van der Waals contacts. To distinguish the nature of these interactions, the eigenvalues (λ_1_ ≤ λ_2_ ≤ λ_3_) of the Hessian matrix of ρ are examined. The quantity sign (λ_2_)ρ is obtained by multiplying the electron density by +1 if λ_2_ > 0 or –1 if λ_2_ < 0. This function distinguishes between:

- Attractive interactions (λ_2_ < 0, blue)
- Weak dispersion interactions (λ_2_ ≈ 0, green)
- Repulsive or steric regions (λ_2_ > 0, red)

The iso-surfaces shown in Figure 4 were generated from the calculated electron density and plotted using Multiwfn, a post processing software to analyze wavefunctions [31]. Each colored surface represents a three-dimensional region in space where these interactions occur—it is not a planar cut or projection, but a true spatial mapping of the interaction field around the molecule.

By default, NCI plots only display noncovalent interactions such as delocalized π-electron, σ···π and π···π staking interactions, attractive van der Waals, hydrogen bond, and excluding any covalent interactions. Figure S4 shows the position of RDG iso-surfaces of benzene and phenol monomers at the center of their aromatic rings (labelled as A), indicating delocalized π-electron of carbon atoms of the ring. Anisole and guaiacol monomers provide richer visualization RDG iso-surfaces with partial steric repulsion C···C atoms and partial attractive van der Waals interaction C···H atoms (labels G and I). Guaiacol monomer also exhibits additional steric repulsion (O···O, label J) and hydrogen bond (O···H, label L) between hydroxyl and methoxy groups. These J- and L-type interactions are only observable in guaiacol monomers with the anti–syn conformation, where the hydrogen atom of the hydroxyl group points towards the oxygen atom of the methoxy group. Based on the NCI plots of the monomers, we now proceed to examine and analyze the NCI plots of the dimers, which exhibit more complex RDG profiles, as shown in Figure 4. Beginning with benzene, the NCI plots of the benzene dimer reveal two additional noncovalent interactions: (B) CH···π stacking interactions and (C) attractive van der Waals interactions of C···H, observed between neighboring benzene monomers in the T-shaped (PerT) configuration. In contrast, for the parallel displaced (ParO) configuration of the benzene dimer, π···π stacking interactions (B) and attractive C···C or C···H van der Waals interactions are observed (see Figure S5a).

Lignin monomers with hydroxyl and/or methoxy groups, such as phenol, anisole, and guaiacol, have greater tendency to form hydrogen bonds with their identical neighbors. The bonding strength of the hydrogen bonds depends on the dimer configuration and the number of hydrogen bonds formed. For example, the phenol dimer in the edge-to-edge configuration exhibits two distinct hydrogen-bonding interactions: a strong hydroxyl–hydroxyl (E) hydrogen bond and a moderate aromatic–hydroxyl (D) hydrogen bond (see Figure 4b). Previous infrared spectroscopy and computational studies reported only the hydrogen bonds via hydroxyl–hydroxyl interaction observed in phenol dimer [26], likely because the phenols were modeled in a linear configuration without aromatic overlap, which precludes the aromatic–hydroxyl interaction. The guaiacol dimer also exhibits hydrogen bonds formed through hydroxyl–methoxy (M) interactions with moderate bonding strength. An additional attractive van der Waals interaction in the guaiacol dimer, arising from aromatic–hydroxyl contact (N), is evident in the NCI plot. On the other hand, no hydrogen bond is observed in the anisole dimer; only an attractive aromatic–methoxy (K) interaction is present. However, the anisole dimer in the ParO1 configuration (see Figure S5c) exhibits a hydrogen bond through a methoxy–methoxy (H) interaction. All noncovalent interactions observed in the four lignin derivatives are summarized in Table 1, along with their corresponding descriptions. In the following paragraph, we will further elaborate on how hydrogen-bonds formed in phenol, anisole, and guaiacol dimers are closely related to peak shift of O–H stretching, O–H bending, C-H stretching and C-H bending modes revealed in their IR spectra with respect to their monomers.

### 2.4. IR Spectra of Dimers

**Figure 5:**
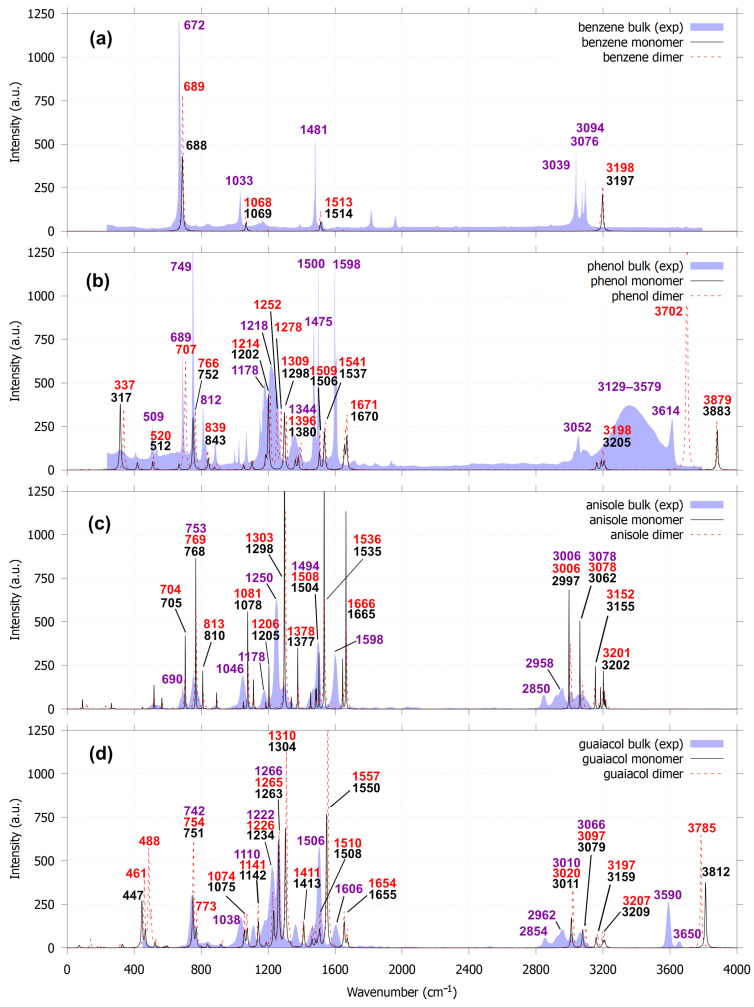
Comparison of calculated IR spectra for the most stable monomers and dimers for benzene (**a**), phenol (**b**), anisole (**c**) and guaiacol (**d**), based on the lowest Δ$G_{rel}^{{}^{\circ}}$ values, with their respective experimental IR spectra obtained from the NIST Chemistry WebBook [42].

The introduction of empirical approaches for incorporating van der Waals interactions into practical calculations by Grimme [41], particularly in DFT, followed by the development of the noncovalent interaction (NCI) method [29,30], has enabled detailed investigation of long-range interactions. Consequently, the assignment of IR peaks associated with noncovalent interactions has become feasible. For the benzene dimer with the T-shaped (PerT) configuration, the NCI RDG plot can visually provide the location of the isosurface which represents CH···π stacking. We observed a very small shift of the C–H out-of-plane bending mode from 688 to 689 cm^−1^; the C–H in-plane bending modes at 1069 and 1514 to 1068 and 1513 cm^−1^, respectively; and the aromatic C–H stretching mode from 3197 to 3198 cm^−1^ with respect to its monomer (Figure 5). For parallel displaced (ParO) benzene dimer, IR peak shifts are observed only for the out-of-plane C–H bending mode (Figure S6a). Compared to the experimental IR spectrum of benzene measured in solution [42], the unscaled calculated IR peaks are positioned slightly to the right of the observed benzene IR peaks. The calculated and observed peaks differ in both intensity and position due to concentration and also solvent effects [43,44].

Phenol, anisole, and guaiacol dimers exhibit more pronounced spectral changes. For the phenol dimer, the free O–H stretching band shifts from 3883 to 3879 cm^−1^, and a new bound O–H stretching mode appears at 3702 cm^−1^, accompanied by a bound O–H bending mode at 707 cm^−1^, indicating strong hydrogen bonds are formed in phenol dimer (Figure 4b, label E). However, another predicted bond (label D) is not evident in the IR spectra. A previous IR-DFT study shows that phenol dimers in a linear configuration can exhibit both free and bound O–H stretching modes [26]. Experimental IR spectra of phenol in solution [42] reveal a broad intermolecular hydrogen-bonding feature in the 3129–3579 cm^−1^ region, corresponding to the bound O–H stretch. As the phenol concentration increases, this bound O–H stretching band shifts progressively to lower frequencies. The wide O–H stretching region indicates that phenol solutions contain multiple molecular aggregates, including dimers, trimers, tetramers, and larger clusters. Based on these observations, we argue that phenol dimers should display both free and bound O–H stretching modes, consistent with previous IR spectroscopy and DFT study, the experimental IR spectrum of phenol solution, and the edge-to-edge phenol dimer identified in this work.

Guaiacol dimers also form hydrogen bonds. In the guaiacol dimer, the hydrogen bond formed between adjacent hydroxyl and methoxy groups exhibits an O–H stretching vibration at 3785 cm^−1^, representing a red shift from 3812 cm^−1^. A previous IR/UV double-resonance and DFT study of guaiacol dimers [24] reported a strong band assigned to the asymmetric O–H stretching mode at 3559 cm^−1^ and concluded that the dimer structure consists of two guaiacol units interacting exclusively through hydrogen bonding between their hydroxyl and methoxy groups, without aromatic ring overlap. The experimentally observed IR spectrum of guaiacol in solution (Figure 5) shows two O–H stretching features at 3590 and 3650 cm^−1^, which are attributed to asymmetric and symmetric O–H stretching modes. In our study, the guaiacol dimer similarly exhibits two degenerate O–H stretching modes at 3783 cm^−1^ (symmetric) and 3785 cm^−1^ (asymmetric); however, these modes are effectively indistinguishable due to their very small frequency separation.

The most stable anisole dimer based on the Δ$G_{rel}^{{}^{\circ}}$ values does not form a hydrogen bond, whereas the most stable structure at 0 K contains a single hydrogen bond (structure H, Figure S5c). The vibrational modes in the 2800–3200 cm^−1^ region are dominated by C–H stretching motions of the methoxy group, as supported by both theoretical and experimental IR spectra. However, unlike benzene, phenol, and guaiacol, the average peak intensities of the anisole dimer are approximately half those of the monomer. Similar effects have been reported in 1D and 2D infrared spectroscopy studies of amyloid fibers [45] and in a combined IR-DFT study of dimethylformamide (DMF) interaction with phenol derivatives [46]. This decrease in intensity arises from changes in the electronic density (ρ) distribution and the change in the dipole moments (*μ*) when long-range intermolecular interactions develop between the approaching monomers, as well as from the influence of the methoxy substituent. To further elucidate this behavior, it is necessary to examine the fundamental origins of infrared peak positions, intensities, and bandwidths.

Infrared peak positions are governed by the force constants of the interacting atoms—arising from ionic or covalent bonding—and by the total mass of the atoms involved in a given vibrational mode [47,48]. In contrast, infrared peak intensities are influenced by the molecular concentration in the sample and by changes in the dipole moment. The IR wavenumber ($\tilde{\nu }$) for a specific vibrational mode can be expressed as

(2) $$\tilde{\nu }=\frac{1}{2\pi c}\sqrt{\frac{k}{\mu }}$$

where *c*, *k*, and *μ* refer to the speed of light, the force constant of the bond (N/m) and the reduced mass of the atoms in molecules, respectively. The IR wavenumber is directly proportional to the square root of the Hessian matrix eigenvalues and the Hessian matrix itself depends on the electronic energy curvature, density response to nuclear displacements, and thus the electron density (ρ) [49]. Therefore, the IR frequency reflects how the electron density resists distortion as the interacting atoms vibrate and their chain relations can be approximated as

(3) $$\tilde{\nu \;}\propto \;\sqrt{k}\propto \;\sqrt{\frac{\partial^{2}E}{\partial x_{i}\partial x_{j}}}\propto \sqrt{\frac{\partial }{\partial x_{i}}\left(\int \rho (r)\frac{\partial V_{ext}}{\partial x_{j}}d^{3}r\right)}$$

Accordingly, the infrared peak shifts arise from changes in the electron density (ρ) or its spatial gradient ∇ρ(r) caused by weak interactions between neighboring molecules—as shown by the NCI plots in Figure 4—which can indirectly modify the force constants of the interacting atoms. These perturbations shift the vibrational frequencies to lower (red shift) or higher (blue shift) energies. This mechanism accounts for the IR frequency changes observed for benzene, phenol, anisole and guaiacol dimers relative to their monomers in Figure 5. This behavior is expected because both the reduced density gradient (s) in the NCI plots and the IR frequencies ($\tilde{\nu }$) are highly sensitive to the underlying electron density (ρ) distribution of the monomers and dimers. While partial atomic charges do not capture the full detail of electron density (ρ), they nonetheless provide a useful indicator of the electron-density redistribution upon dimerization. As shown in Figure S7, the partial charges of the monomer units within each dimer differ from those of the isolated monomers. This demonstrates that dimer formation induces interaction-driven adjustments in electron density within each monomer, thereby altering the IR peak positions and intensities of the dimers relative to their monomeric counterparts.

The vibrational modes of a molecule are observable in the infrared spectrum when the molecule can absorb IR radiation and undergoes a vibrational motion in which the change in dipole moment with respect to atomic displacement is non-zero (∂μ/∂Q≠0, where *μ* and Q represent the dipole moment and atomic coordinates, respectively). Therefore, the IR peaks intensity depends on the dipole-moment derivative of each normal mode, which can be approximated as [47,48].

(4) $$I\;\propto \;{\left|\frac{\partial \mu }{\partial Q}\right|}^{2}$$

In the anisole dimer, opposing components of the dipole-moment derivatives can interfere destructively (antisymmetric coupling), thereby reducing the intensity of the associated vibrational transitions. Conversely, aligned components lead to constructive (symmetric) interference and enhanced intensity. For the most stable anisole dimer configuration, the methoxy groups promote strong destructive interference, substantially attenuating many vibrational bands. The remaining vibrational modes assignments are summarized in Table S2.

### 2.5. Steric Repulsion-Dependent Dihedral Angle of 3,3′-Dimethoxy-1,1′-Biphenyl-2,2′-Diol

**Figure 6:**
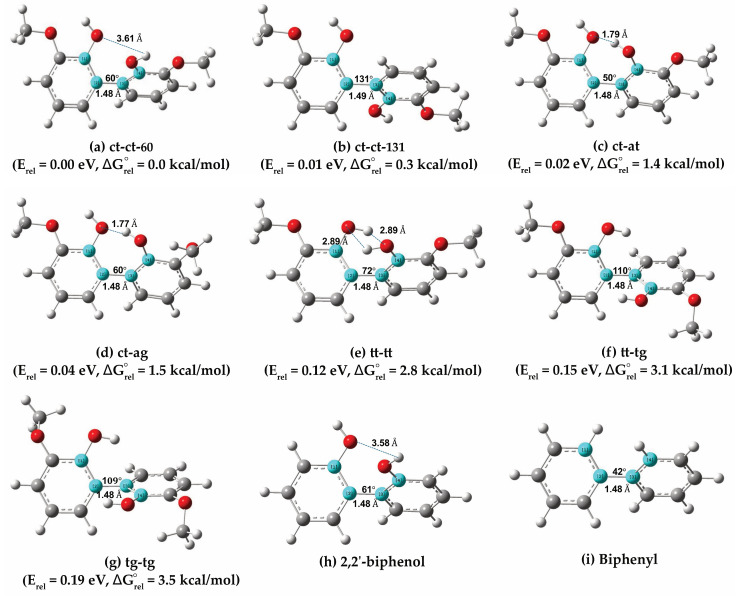
Structures of 3,3′-dimethoxy-2,2′-biphenyl-diol with ct-ct-60 (**a**), ct-ct-131 (**b**), ct-at (**c**), ct-ag (**d**), tt-tt (**e**), tt-tg (**f**) and tg-tg (**g**) configurations. The optimized 2,2’-biphenol (**h**) and biphenyl (**i**) structures are provided as reference. ct, at, ag, tt, tg and ag labels corresponding to cis–trans, anticlinal–trans, anticlinal–gauche, trans–trans, trans–gauche, and anticlinal–gauche, respectively. White, grey, and red atoms represent hydrogen, carbon, and oxygen atoms, respectively. Four atoms colored in light blue marked the dihedral angle between two aromatic rings.

Guaiacyl moiety, a lignin derivative, serves as a precursor for biofuels, flavorings, deodorants, antiseptics, sedatives and antioxidants. Enzymatic oxidation of guaiacol produces dimers, trimers, and tetramers via C–C or C–O coupling [50]. Among the four guaiacyl dimers identified by NMR—3,3′-dimethoxy-1,1′-biphenyl-4,4′-diol; 3,3′-dimethoxy-1,1′-biphenyl-2,4′-diol; 3,3′-dimethoxy-1,1′-biphenyl-2,2′-diol; and 3′-methoxy-1′-(methoxyphenoxy) phenol—the first three are linked via C–C bonds, while the last features an ether linkage [22,50]. In this study, we focus on 3,3′-dimethoxy-1,1′-biphenyl-2,2′-diol, where the hydroxyl groups are positioned proximally, leading to significant long-range interactions such as steric repulsion. To identify the most stable conformations, we examined ten combinations of aromatic rings (Table S1) and obtained seven optimized conformers, as shown in Figure 6. The most stable configuration is ct-ct-60 (dihedral angle 59.6°), with guaiacol units in an anti–syn conformation, followed by the structurally similar ct-ct-131 (dihedral angle 130.6°). The ct-at (anti–anti) and ct-ag (gauche–anti) conformations rank third and fourth, with energy differences of only a few tens of meV. Configurations with anti–anti (tt-tt), gauche–anti (tg-tg), or mixed (tt-tg) conformations exhibit slightly higher energies (~100 meV). The stability trend is also consistent with their Δ$G_{rel}^{{}^{\circ}}$ values, where ct-ct-60 is the most stable configuration while tg-tg is the least stable. Based on hydroxyl orientations, these structures can be classified into two groups: (i) hydroxyl hydrogens pointing in opposite or similar directions (ct-ct-60, ct-ct-131, ct-at, ct-ag) and (ii) hydroxyl hydrogens facing each other (tt-tt, tt-tg, tg-tg). Similarly to biphenyl and 2,2’-biphenol, the stability of guaiacyl dimers is primarily dictated by steric repulsion between neighboring atoms or functional groups near the C–C linkage, which in turn defines the dihedral angles between the aromatic rings. The results indicate that neither small nor large dihedral angles necessarily correspond to the lowest-energy structures. Notably, the most stable configuration of 3,3′-dimethoxy-1,1′-biphenyl-2,2′-diol retains a C–C bond length of 1.477 Å, closely matching that of 2,2’-biphenol and biphenyl. Its dihedral angle about the C–C linkage is also very similar to that of 2,2′-biphenol.

**Figure 7:**
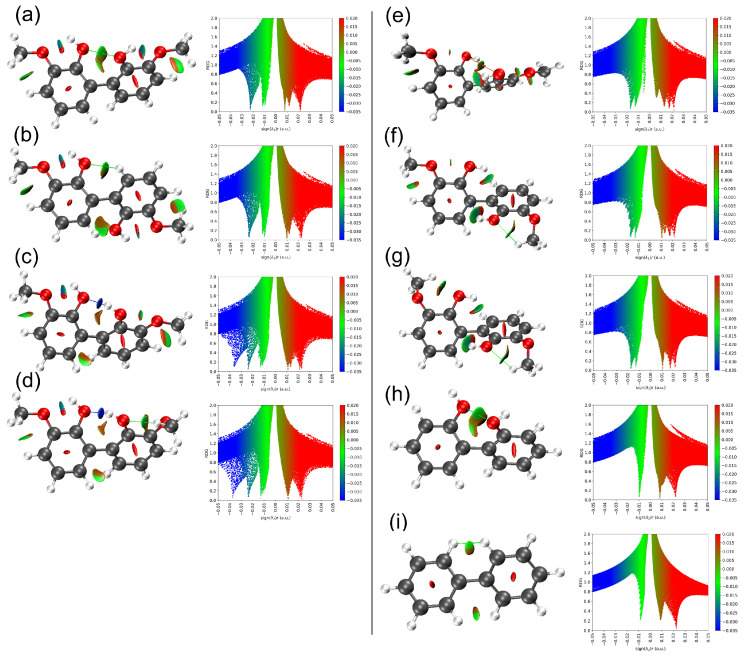
NCI plots of the reduced density gradient (RDG) iso-surfaces (left) and its associated 2D (s, *ρ*) diagram (right) for 3,3′-dimethoxy-1,1′-biphenyl-2,2′-diol with ct-ct-60 (**a**), ct-ct-130 (**b**), ct-at (**c**), ct-ag (**d**), tt-tt (**e**), tt-tg (**f**) and tg-tg (**g**) configurations. The NCI plots for 2,2’-biphenol (**h**) and biphenyl (**i**) are also shown as reference. The surfaces are colored on a blue-green-red scale according to values of sign (λ_2_)*ρ*, ranging from −0.05 to +0.05 au. Blue, green and red colors denote strong attractive interaction, weak interaction, and strong nonbonded overlap, respectively.

To further elucidate the influence of long-range interactions, such as steric repulsion and hydrogen bonding, near the C–C linkage in dictating the dihedral angle, noncovalent interaction (NCI) plots were analyzed (Figure 7). The NCI plots of 3,3′-dimethoxy-1,1′-biphenyl-2,2′-diol, 2,2’-biphenol, and biphenyl clarify the relationship between these interactions and the observed dihedral angles. There is a common conjecture that the dihedral angle between the two aromatic rings never reaches zero because coplanarity would significantly increase steric repulsion between hydroxyl groups or neighboring atoms (as in biphenyl). To minimize this repulsion, one ring rotates about the C–C linkage axis. In the ct-ct-60 configuration, a 59.6° rotation reduces steric hindrance and yields an O···O separation of 2.894 Å, indicative of a weak attractive interaction (Figure 7a). In 2,2′-biphenol, rotation increases slightly to 61.5° to further alleviate steric repulsion, consistent with its marginally shorter O···O distance of 2.885 Å (Figure 7h). Notably, no NCI isosurface appears between adjacent hydrogen atoms in these configurations, likely due to the longer H···H separations (2.744–2.764 Å) compared with biphenyl (2.420 Å). In contrast, two NCI isosurfaces are evident near the C–C linkage in the ct-ct-130 configuration (Figure 7b), indicating O···H interactions between hydroxyl groups and neighboring hydrogen atoms at estimated distance of 2.595 Å. These observations suggest that dihedral angles between approximately 60° and 130° effectively minimize steric repulsion in the guaiacyl dimer for ct-ct configurations.

When one hydrogen atom of the hydroxyl groups in the C–C–linked dimer reorients toward a neighboring hydroxyl group, a strong hydrogen bond forms with the adjacent oxygen atom, as indicated by the additional deep-blue region in the 2D NCI plots (Figure 7c,d). Consequently, the dihedral angle decreases to ~49.5°, as the hydrogen bond partially offsets steric repulsion between adjacent hydroxyl groups. This interaction also slightly elongates the inter-ring C–C bond by 0.003 Å. The corresponding O···H distances are 1.788 Å (ct-at) and 1.771 Å (ct-ag). The transition from anti–anti (ct-at) to anti–gauche (ct-ag) does not significantly affect the dihedral angle because the hydroxyl–methoxy O···H interaction is weaker than the hydroxyl–hydroxyl counterpart. In contrast, when both hydroxyl hydrogens face each other, steric repulsion intensifies, forcing additional ring rotation and resulting in larger dihedral angles, as observed in the tt-tt, tt-tg and tg-tg configurations. In the tt-tt conformation, a dihedral angle of ~72° represents the minimal value required to reduce steric repulsion between hydroxyl groups, as evidenced by a small red (repulsive) iso-surface in the O···O region (3.256 Å, Figure 7e). Weak O···H interactions at 2.890 Å are likely responsible for this repulsive feature, as they draw the hydroxyl groups closer, consistent with observations in the ct-at and ct-ag conformations, even though no explicit NCI iso-surface confirms these interactions. However, comparison of 2D NCI plots for the tt-tt, tt-tg and tg-tg configurations reveals a narrow green line near (λ_2_)ρ ≈ 0 which exist only in tt-tt, indicating that repulsive O···O interactions diminish as a new hydroxyl–methoxy hydrogen bonding disrupts long-range hydroxyl–hydroxyl interactions between the aromatic rings. This interpretation is supported by the increase in dihedral angle from 72° to ~109°, associated with a 90° methoxy rotation during the transition from tt-tt to tt-tg. Further transition from tt-tg to tg-tg does not increase the dihedral angle further since the long-range hydroxyl–hydroxyl interactions have been broken in tt-tg configuration.

**Figure 8:**
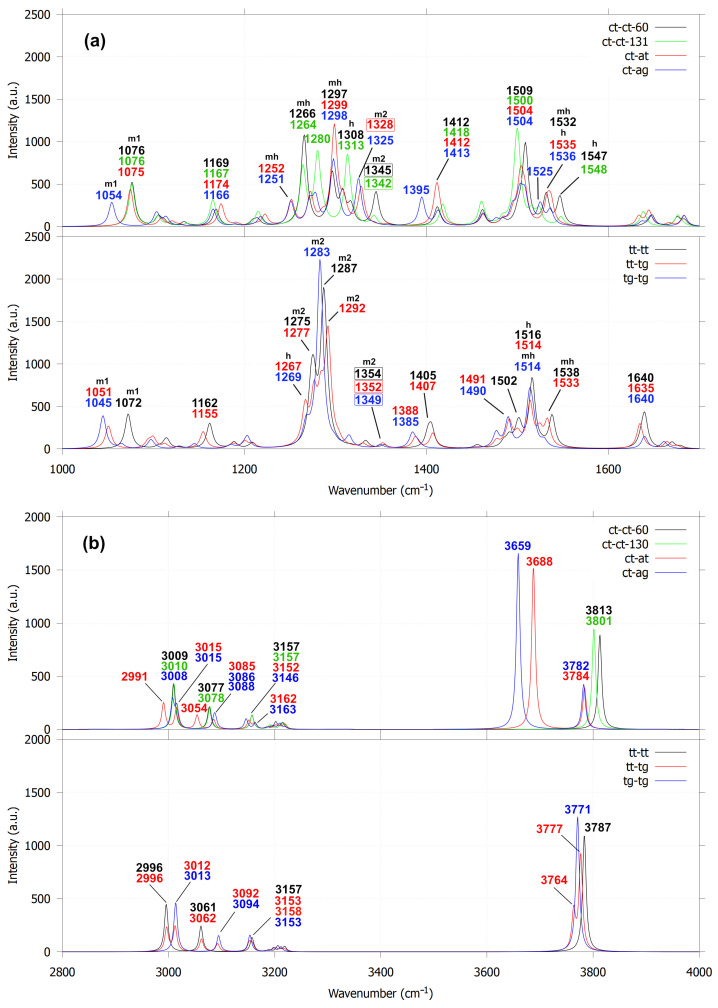
Calculated IR spectra for 3,3′-dimethoxy-2,2′-biphenyl-diol for the frequency ranges at 1000–1700 cm^−1^ (**a**) and 2800–4000 cm^−1^ (**b**). The labels m1, m2, h and mh represent the stretching modes of O-CH_3_, C-OCH_3_, C-OH, and a combination of C-OCH_3_ and C-OH, respectively.

**Figure 9:**
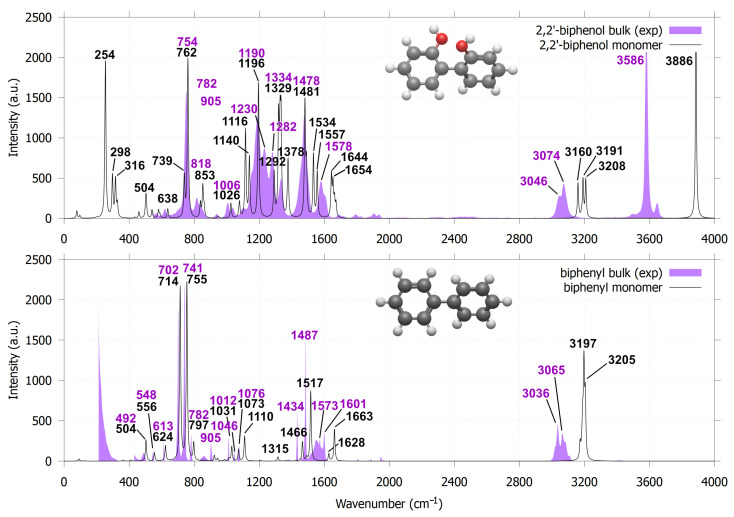
Calculated IR spectra for the most stable monomers for 2,2’-biphenol and biphenyl, with their respective experimental IR spectra obtained from the NIST Chemistry WebBook [42].

The sensitivity of dihedral angles in 3,3′-dimethoxy-1,1′-biphenyl-2,2′-diol to the orientations of hydroxyl and methoxy groups is reflected in their IR spectra through peak shifts and the emergence of new vibrational features. The first group of guaiacyl dimers exhibits two distinct C–C linkage stretching modes in the 1300–1400 cm^−1^ region, which are absent in dimers lacking C–C coupling. As shown in Figure 8a, these modes appear at 1345, 1342, 1328 and 1303 cm^−1^ (see the numbers within the boxes), respectively, for the ct-ct-60, ct-ct-130, ct-at, ct-ag conformations. The 1303 cm^−1^ peak (ct-ag) is obscured by a stronger band at 1298 cm^−1^, attributed to overlapping C–OCH_3_ and C–OH stretching vibrations. For comparison, 2,2′-biphenol and biphenyl were used as reference molecules because their IR spectra are well established. Biphenyl exhibits C–C (linkage) stretching modes at 1321 and 1547 cm^−1^ (Figure 9). The 1321 cm^−1^ mode is IR-inactive, whereas the 1547 cm^−1^ mode is obscured by an intense in-plane C–H bending band at 1517 cm^−1^. This is consistent with experimental IR spectra of biphenyl solutions, which show no peak near 1321 cm^−1^ and display a band at 1487 cm^−1^ attributed to in-plane C–H bending, slightly red-shifted from the calculated value. In 2,2′-biphenol, the C–C (linkage) stretching mode appears at 1328 cm^−1^ (IR-active) and the experimental IR spectrum suggests a corresponding feature near 1282 cm^−1^, likely arising from mixed C–C (ring/linkage) and in-plane C–H vibrational character.

Similarly, C–C (linkage) stretching modes in the second group (tt-tt, tt-tg, tg-tg) are barely detectable for the same reasons. Their corresponding peaks (at 1354, 1352 and 1349 cm^−1^) lie close to those of the ct-ct-60 and ct-ct-130 configurations. The limited spectral shifts in this group arise from the absence of hydroxyl reorientation and hydrogen bonding, unlike in the first group. This distinction also influences their overall IR profiles: the C–OCH_3_(m2) stretching modes (1200–1400 cm^−1^) in the second group display nearly twice the intensity and narrower bands compared to the first group, where broader peaks result from mixed C–OCH_3_ and C–OH (mh) and C–OCH_3_ (m2) stretching contributions.

In the 2900–4000 cm^−1^ region (Figure 8b), the IR spectra exhibit three distinct groups of vibrations: C–H stretching modes of methoxy groups (2900–3170 cm^−1^) and aromatic rings (3170–3250 cm^−1^) and O–H stretching modes of hydroxyl groups (3600–3800 cm^−1^). For the first group of conformations, ct-ct-60 and ct-ct-131 each display a single O–H stretching band at 3813 and 3801 cm^−1^, respectively. In contrast, ct-at and ct-ag exhibit two types of O–H stretching: free O–H modes at 3784 and 3782 cm^−1^, comparable to the O–H stretching of the non–C–C–linked guaiacol dimer (3785 cm^−1^, Figure 5d) and bound O–H modes associated with hydrogen bonding at 3688 cm^−1^ (ct-at) and 3659 cm^−1^ (ct-ag). These results suggest that ct-at forms a stronger hydroxyl–hydroxyl hydrogen bond near the C–C linkage, whereas ct-ag involves weaker hydroxyl–methoxy interactions. The second group (tt-tt, tt-tg, tg-tg) exhibits only free O–H stretching modes. While variations also appear in the methoxy C–H stretching region, the O–H stretching profiles provide a more reliable means of distinguishing the different configurations of 3,3′-dimethoxy-1,1′-biphenyl-2,2′-diol. Additional vibrational assignments are summarized in Table S3. In comparison to 2,2′-biphenol and biphenyl (Figure 9), the vibrational features in the 2900–3250 cm^−1^ range correspond exclusively to symmetric and asymmetric in-plane C–H stretching of the aromatic rings. The IR peaks associated with these vibrational modes exhibit higher and sharper intensities than the corresponding peaks observed for 3,3′-dimethoxy-1,1′-biphenyl-2,2′-diol, for which they appear only as a small bump in the IR spectrum. The presence of these in-plane aromatic C–H stretching modes is further supported by the experimental IR spectra of 2,2′-biphenol and biphenyl, which show peaks in the 3000–3100 cm^−1^ region.

Based on the above discussion, several features distinguish 3,3′-dimethoxy-1,1′-biphenyl-2,2′-diol from both non–C–C–linked guaiacol dimers (in parallel displaced) and biphenyl are:

1. In its most stable conformations (ct-ct-60 and ct-ct-131), the guaiacyl moiety exhibits distinct C–C (linkage) stretching bands in the 1300–1400 cm^−1^ region of the IR spectrum.
2. In ct-at and ct-ag conformations, two types of O–H stretching modes—free and bound hydrogen —appear between 3600 and 3800 cm^−1^.
3. The tt-tt, tt-tg, and tg-tg conformations lack observable C–C or O–H stretching bands but display intense, narrow C–OCH_3_ stretching peaks in the 1200–1400 cm^−1^ range.
4. At 2900–3300 cm^−1^, the IR peaks observed in 3,3′-dimethoxy-1,1′-biphenyl-2,2′-diol correspond to C–H stretching modes of the methoxy groups and the aromatic rings, whereas in 2,2′-biphenol and biphenyl they correspond to C–H stretching modes of the aromatic rings.

## 3. Computational Methods

We performed density-functional theory calculations as implemented in Gaussian 16, Revision C.01 [51] to study the structures and infrared spectra of four lignin derivatives—benzene, phenol, anisole and guaiacol—as monomers and dimers. The calculations employed the APFD functional [52] and 6-311++G(d,p) basis set. The functional provides an empirical dispersion correction term essential for modelling NCI and has successfully modelled the potential energy surface of small hydrocarbon dimers with a comparable accuracy to the rigorous CCSD(T)/aug-cc-pVTZ level of theory [52]. In our previous study, the combination of the functional and basis set has successfully modelled dispersion interactions which are critical for system with NCI [53]. Moreover, the most stable configuration of dimers obtained by the APFD functional were consistent with other functionals which considered NCI (M06-2X) and long-range corrected term (ωB97X-D) as presented in Table S4. The geometry of each molecule (monomers and dimers) was fully optimized to a stationary point followed by a vibrational frequency calculation using the same functional and basis set to (1) ensure that the geometry was true minimum on the potential energy surface, characterized by the absence of imaginary frequencies and (2) obtain the IR spectra and various thermochemical values. Following the geometry optimizations and vibrational frequency calculations, natural bond orbital (NBO) analysis and noncovalent interaction (NCI) analysis were conducted. The former was using NBO version 3.1 [54], while the latter was using Multiwfn, version 3.7 [31].

## 4. Conclusions

This study demonstrates the effectiveness of noncovalent interaction (NCI) analysis in elucidating long-range molecular interactions within four lignin derivatives (benzene, phenol, anisole and guaiacol) and a representative guaiacyl dimer, 3,3′-dimethoxy-1,1′-biphenyl-2,2′-diol. Unlike conventional approaches that rely on inferred interaction patterns, NCI analysis provides a direct visualization of σ···π and π···π stacking, hydrogen bonding, van der Waals attractions and steric repulsion between adjacent atoms or oxygenated functional groups. These detailed insights enable a more comprehensive understanding of how such noncovalent interactions govern the IR spectral behavior of lignin-based systems. Both the NCI plots and the IR peak positions and intensities are functions of the electron density (ρ). The long-range weak interactions present upon dimer formation induce electron redistribution within each monomer unit, as evidenced by changes in their partial charges compared with those of the isolated monomers. Furthermore, NCI plots effectively explain the dependence of dihedral angles in 3,3′-dimethoxy-1,1′-biphenyl-2,2′-diol on hydroxyl reorientation, revealing how this adjustment mitigates steric repulsion and stabilizes the molecular configuration.

## Figures and Tables

**Table 1:** List of interaction types of NCI plots observed in monomers and dimers of Lignin derivatives.

Labels	Interaction Geometry	Interaction Description	Type of Interaction
A	Single aromatic ring (self-interaction)	Opposing C···C atoms (in para position)	delocalized π-electron
B	Parallel offset/Edge-to-edge	C···π, CH···π and π···π	σ···π and π···π staking interactions
C	Parallel offset/Edge-to-edge	C···C and/or C···H	attractive van der Waals
D	Parallel offset/Edge-to-edge	O···H (aromatic–hydroxyl)	H-bond
E	Edge-to-edge	O···H (hydroxyl–hydroxyl)	strong H-bond
F	Parallel offset	O···H (hydroxyl–hydroxyl)	H-bond
G	Single aromatic ring (self-interaction)	Opposing C···C atoms (aromatic–methoxy)	steric repulsion
H	Parallel offset	O···H (methoxy–methoxy)	H-bond
I	Single aromatic ring (self-interaction)	C···H (aromatic–methoxy)	attractive van der Waals
J	Single aromatic ring (self-interaction)	Opposing O···O atoms (hydroxyl–methoxy)	steric repulsion
K	Parallel offset	C···O and/or C···H (aromatic–methoxy)	attractive van der Waals
L	Single aromatic ring (self-interaction)	O···H (hydroxyl–methoxy)	strong H-bond
M	Parallel offset	O···H (hydroxyl–methoxy)	H-bond
N	Parallel offset	C···O (aromatic–hydroxyl)	attractive van der Waals

## Data Availability

The data supporting the conclusions of this article are available at https://doi.org/10.5281/zenodo.17834901 (accessed on 1 December 2025).

## Supplementary Materials

The following supporting information can be downloaded at: https://www.mdpi.com/article/10.3390/molecules30244694/s1, Figure S1: All possible dimer configurations considered in this study.; Figure S2: Electrostatic potential maps for benzene (a), anisole (b), phenol (c) and guaiacol (d) monomers.; Figure S3: Stability order of each dimer configuration according to their relative enthalpy change (Δ$H_{rel}^{{}^{\circ}}$) and Gibbs free energy change (Δ$G_{rel}^{{}^{\circ}}$). Figure S4: NCI plots of the reduced density gradient (RDG) iso-surfaces (left) and its associated 2D (s, *ρ*) diagram (right) for the most stable benzene (a), phenol (b), anisole (c) and guaiacol (d) monomers based on the lowest $E_{rel}$ values.; Figure S5: NCI plots of the reduced density gradient (RDG) iso-surfaces (left) and its associated 2D (s, *ρ*) diagram (right) for the most stable benzene (a), phenol (b), anisole (c) and guaiacol (d) dimers based on the lowest $E_{rel}$ values.; Figure S6: Comparison of calculated IR spectra for the most stable monomers and dimers for benzene (a), phenol (b), anisole (c) and guaiacol (d), based on the lowest $E_{rel}$ values with their respective experimental IR spectra obtained from the NIST Chemistry WebBook.; Figure S7: Partial charge of each atom on the most stable monomers and dimers for benzene (a), phenol (b), anisole (c) and guaiacol (d).; Table S1: All possible and stable conformers of 3,3′-dimethoxy-1, 1′-biphenyl-2,2′-diol; Table S2: Calculated vibrational frequency (cm^−1^) of monomers and dimers of benzene, phenol, anisole and guaiacol and their respective experimental IR spectra obtained from the NIST Chemistry WebBook [55,56,57,58].; Table S3: Calculated vibrational frequency (cm^−1^) of 3,3′-dimethoxy-2,2′-biphenyldiol for six stable conformations.; Table S4: Comparation of relative electronic energy of benzene and guaiacol dimers obtained by APFD (M1), M06-2X (M2) and ωB97X-D (M3).
