# Supplementary material for: The Role of Noncovalent Interactions in the Infrared Spectra of Lignin Model Compounds: A DFT Study
Source: Molecules. 2025 Dec 8;30(24):4694. doi: 10.3390/molecules30244694 (PMC12735836; doi:10.3390/molecules30244694)
Supplement: Supplementary file 1 [file molecules-30-04694-s001.zip › molecules-3965052-supplementary.pdf]

Supporting Information

The Role of Noncovalent Interactions in the Infrared Spectra of Lignin Model Compounds: A DFT Study

Febdian Rusydi [1], Lusia Silfia Pulo Boli [2], Indri Badria Adilina [3], Wahyu Tri Cahyanto [2], Stewart F. Parker [4,*] and Ferensa Oemry [5,*]

[1] Research Center for Quantum Engineering Design, Faculty of Science and Technology, Universitas Airlangga, Jl. Mulyorejo, Surabaya, East Java 60115, Indonesia; rusydi@fst.unair.ac.id

[2] Physics Study Program, Universitas Jenderal Soedirman, Jl. Dr. Soeaparno 61, Purwokerto, Central Java 53123, Indonesia; lusia.silfia@unsoed.ac.id (L.S.P.B.); wahyu.cahyanto@unsoed.ac.id (W.T.C.)

[3] Research Center for Catalysis, National Research and Innovation Agency, KST BJ Habibie, Tangerang Selatan , Banten 15314, Indonesia; indri030@brin.go.id

[4] ISIS Neutron and Muon Source, STFC Rutherford Appleton Laboratory, Chilton, Didcot , Oxfordshire OX11 0QX, UK

[5] Research Center for Quantum Physics, National Research and Innovation Agency, KST BJ Habibie,
Tangerang Selatan , Banten 15314, Indonesia

* Correspondence: stewart.parker@stfc.ac.uk (S.F.P.); ferensa.oemry@brin.go.id (F.O.)

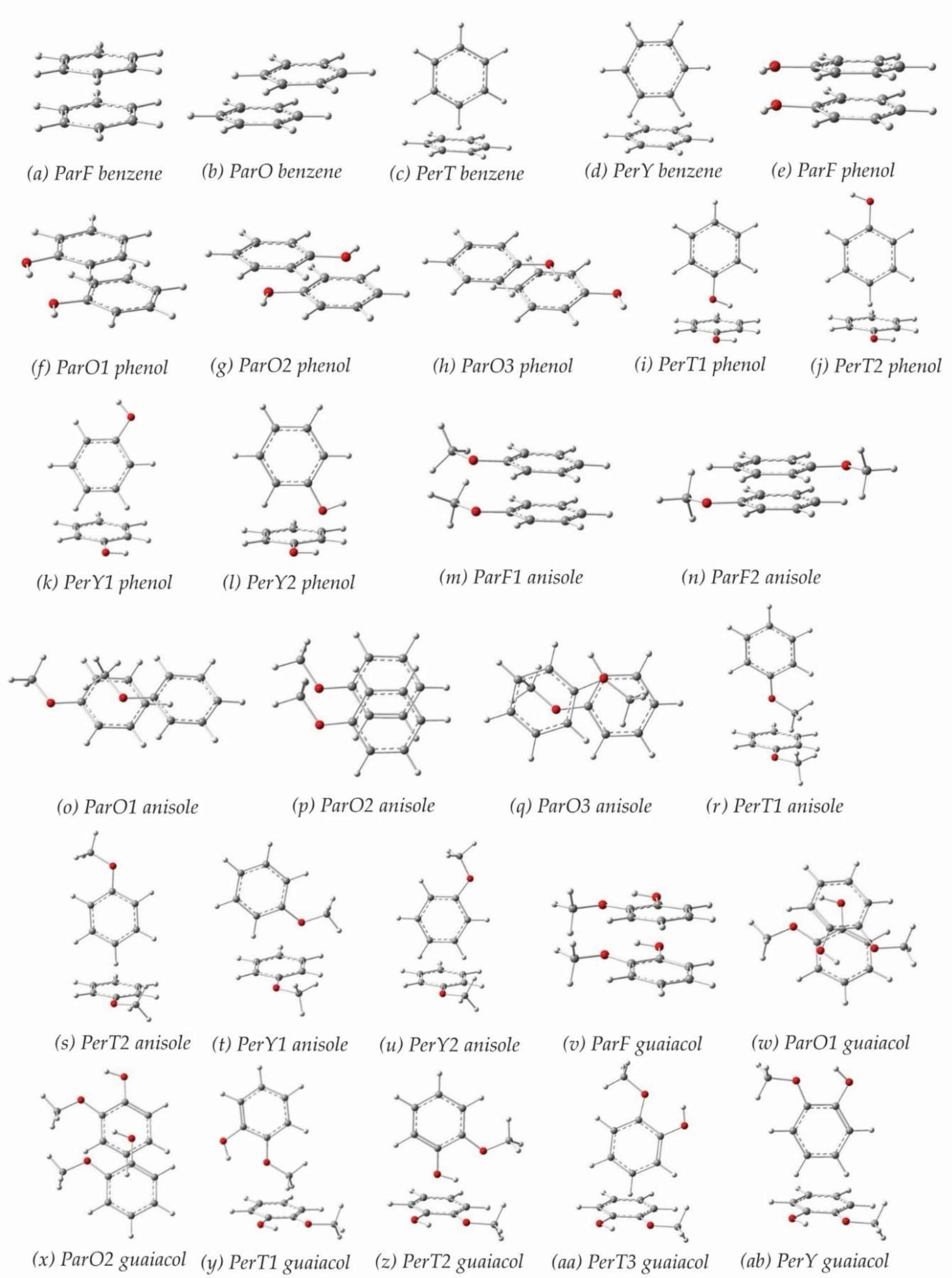

Figure S1. All possible dimer configurations considered in this study. White, grey and red colors are hydrogen, carbon and oxygen atoms, respectively.

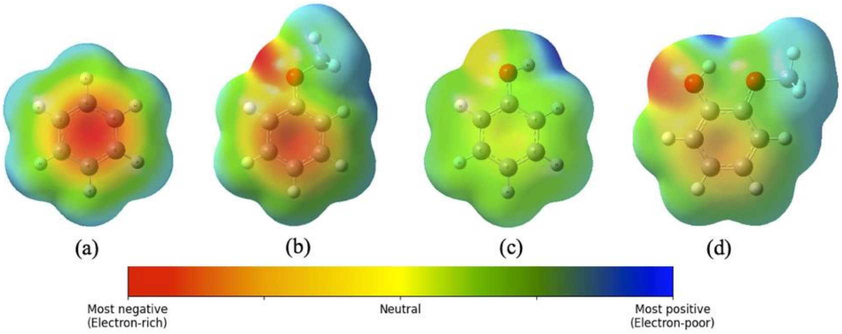

Figure S2. Electrostatic potential maps for benzene (a), anisole (b), phenol (c) and guaiacol (d) monomers.

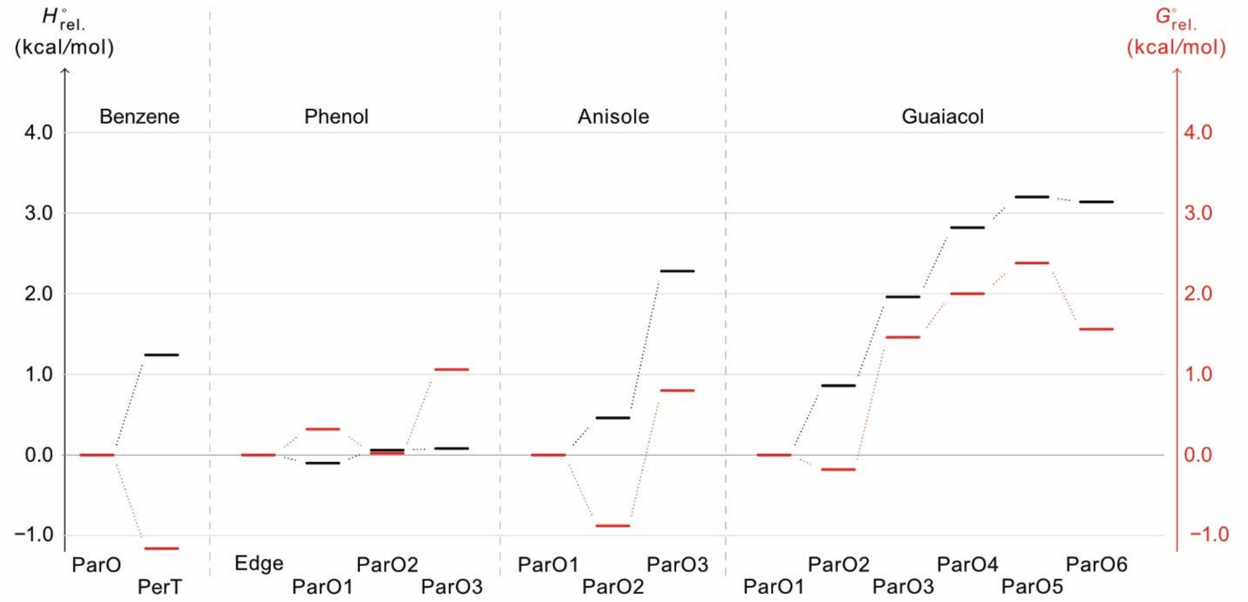

Figure S3. Stability order of each dimer configuration according to their relative enthalpy (H°_{rel}) and Gibbs free energy (G°_{rel}). ParF, ParO, PerT and Edge stand for parallel face-centered, parallel offset, perpendicular T-shaped and edge-to-edge dimer configurations.

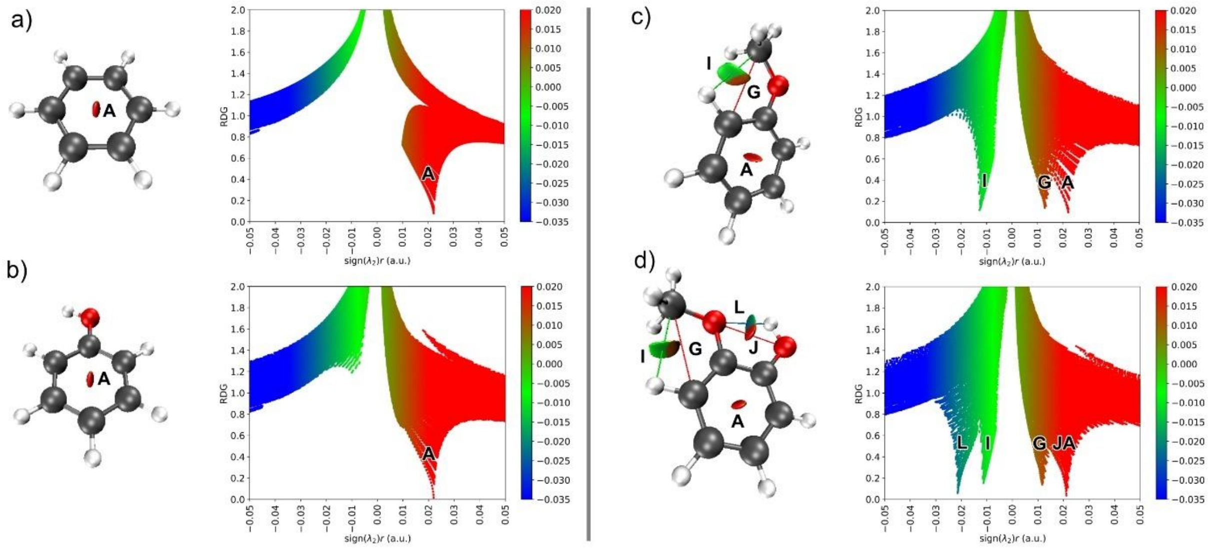

Figure S4. NCI plots of the reduced density gradient (RDG) isosurfaces (left) and its associated 2D (s, ρ) diagram (right) for the most stable benzene (a), phenol (b), anisole (c) and guaiacol (d) monomers dimers based on the lowest E_{rel} values. The surfaces are colored on a blue-green-red scale according to values of sign (λ_2)ρ, ranging from –0.05 to +0.05 au. Blue, green and red colors denote strong attractive interaction, weak interaction and strong nonbonded overlap, respectively.

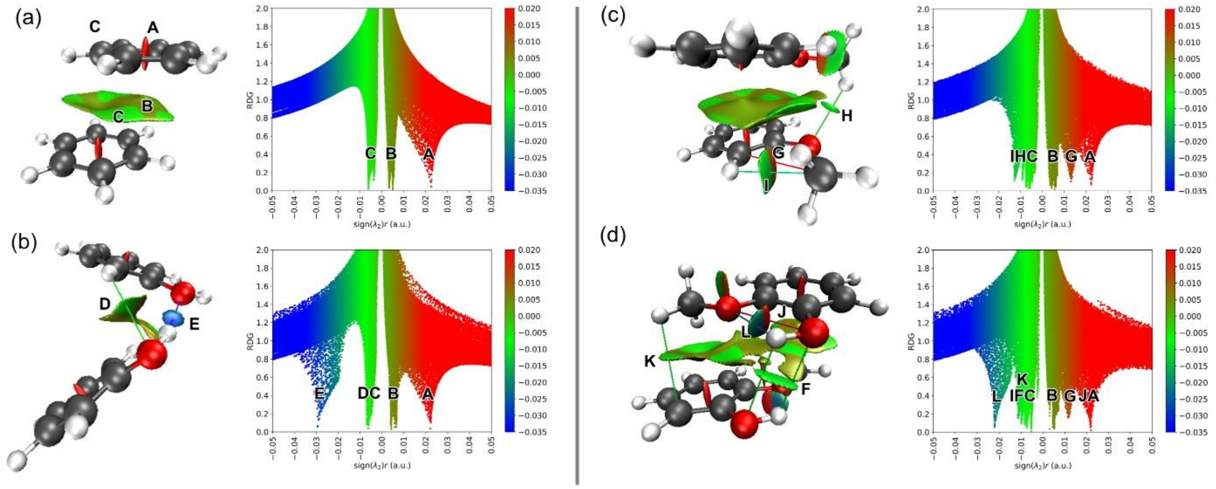

Figure S5. NCI plots of the reduced density gradient (RDG) isosurfaces (left) and its associated 2D (s, ρ) diagram (right) for the most stable benzene (a), phenol (b), anisole (c) and guaiacol (d) dimers based on the lowest E_{rel} values. The surfaces are colored on a blue-green-red scale according to values of sign (λ_2)ρ, ranging from –0.05 to +0.05 au. Blue, green and red colors denote strong attractive interaction, weak interaction and strong nonbonded overlap, respectively.

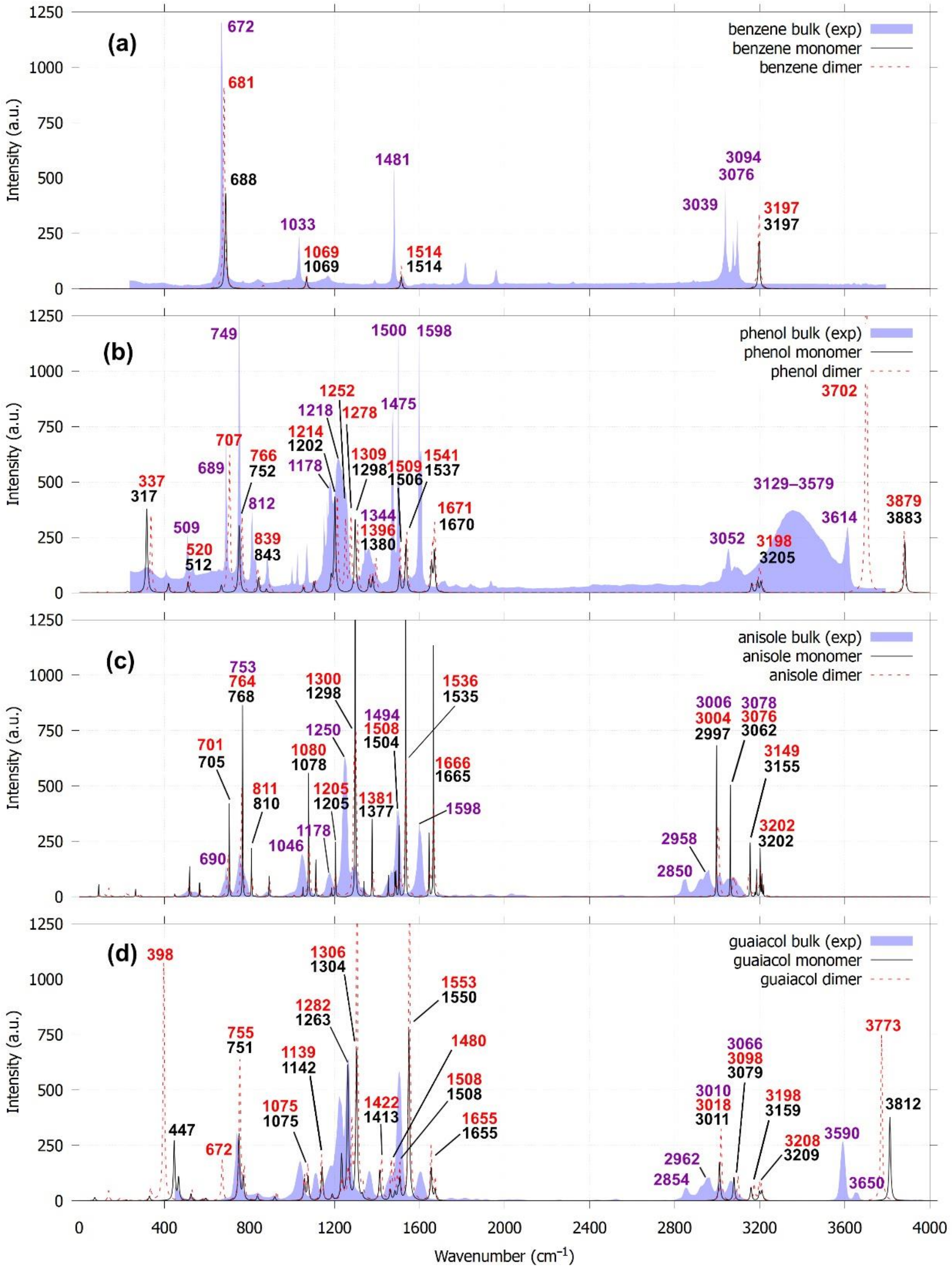

Figure S6. Comparison of calculated IR spectra for the most stable monomers and dimers for benzene (a), phenol (b), anisole (c) and guaiacol (d), based on the lowest E_{rel} values with their respective experimental IR spectra obtained from the NIST Chemistry WebBook [42].

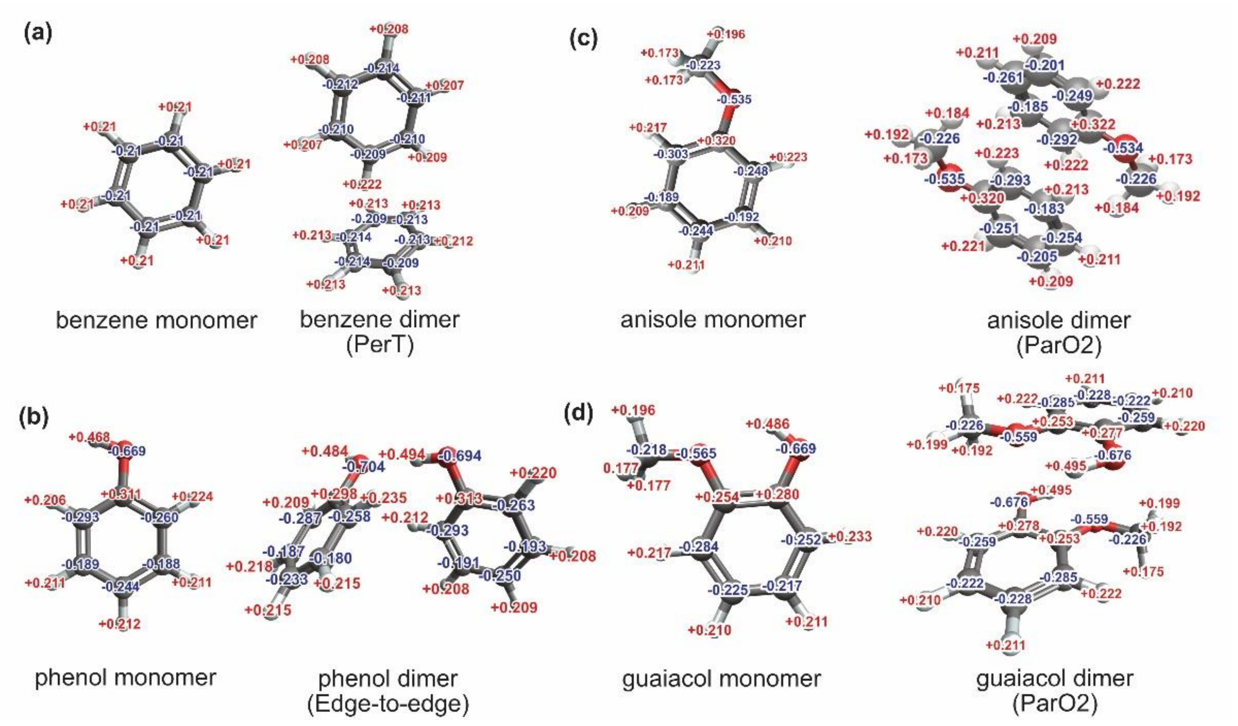

Figure S7. Partial charge of each atom on the most stable monomers and dimers for benzene (a), phenol (b), anisole (c) and guaiacol (d). Red and blue-colored values represent positive and negative partial charges, respectively.

Table S1. All possible and stable conformers of 3,3′-dimethoxy-1, 1′-biphenyl-2,2′-diol. The conformers are identified based on the conformation (cis, gauche, anticlinal, trans) of the O–H and O–CH_3 groups in rings 1 and 2. All possible conformers have a dihedral angle (D) of 0°, except conformer 2 (180°). For conformers 1 and 2, they are distinguished by their dihedral angles after optimization, which were 60 and 131, respectively.

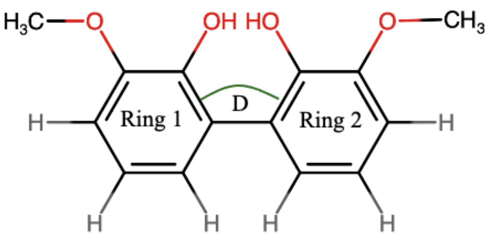

No.	Possible conformers		Stable conformers
	Ring 1	Ring 2	
1.	ct	ct	ct-ct-60
2.	ct	ct	ct-ct-131
3.	ct	tt	ct-at

4.	ct	tg	ct-ag
5.	tt	ct	ct-at
6.	tt	tt	tt-tt
7.	tt	tg	tt-tg
8.	tg	ct	ct-ag
9.	tg	tt	tt-tg
10.	tg	tg	tg-tg

Table S2. Calculated vibrational frequency (cm^{-1}) of monomers and dimers of benzene, phenol, anisole and guaiacol and their respective experimental IR spectra obtained from the NIST Chemistry WebBook.

Mode assignment (This study)	Monomer	Dimer	IR (exp)	Mode assignments (exp)
		Benzene		
δ(C-H) oop	688	689	672	out-of-plane C-H bend
δ(C-H) ip	1069	1068	1033	in-plane C-H bend
δ(C-H) ip	1514	1513	1481	in-plane C-H bend / ring deformation [1]
v(C-H) asym	3197	3198	3039, 3076, 3094	aromatic C-C stretch
		Phenol		
δ(O-H) oop	317	$337^{(SM)}$	509, 539	Ring deformation / skeletal modes
δ(C-H) oop	512	520	689	Out-of-plane C-H bending
δ(C-H) oop + δ(O-H) oop	-	$707^{(SM)}$	749	
δ(C-H) oop	752	766	812, 827, 884	
δ(CCC) ip + *v*($C-OCH_3$)	843	$839^{(SM)}$	1027	C-O stretch (phenolic) and ring C-C stretches / in-plane C-H bends
δ(C-H) ip + δ(O-H)	1202	$1214^{(SM)}$	1070	
δ(C-H) ip + δ(O-H)	-	$1252^{(SM)}$	1152	
v(C-OH)	-	$1278^{(SM)}$	1168	
v($C-OCH_3$)	1298	$1309^{(SM)}$	1178	
v(C-C) + δ(O-H)	1380	$1396^{(SM)}$	1252	
δ(C-H) ip + δ(O-H)	1506	1509	1344, 1359, 1388	in-plane O-H bend / C-O stretch
δ(C-H) ip	1537	1541	1475	C-H bending / ring deformation
v(C-C)	1670	1671	1500, 1598	aromatic C-C stretch / ring mode
v(C-H) asym	3205	3198	3052	aromatic C-H stretch / ring mode
bound *v*(O-H)	-	3702	3129-3579	bound O-H stretch
free *v*(O-H)	3883	3879	3614	free O-H stretch
		Anisole		
δ(C-H) oop	705	$704^{(SM)}$	690	Out-of-plane C-H bending
δ(C-H) oop	768	769	753	Out-of-plane C-H bending
δ(CCC) ip	810	$813^{(SM)}$	1046	in-plane (ring) C-C bends
v($O-CH_3$)	1078	1081	-	-
δ(C-H) ip	1205	1206	1178	in-plane C-H bends
v($C-OCH_3$)	1298	$1303^{(SM)}$	1250, 1294	Aromatic C-O ($Ar-O-CH_3$) stretch
v(C-C)	1377	1378	-	-
γ(CH_3)	1504	$1508^{(SM)}$	-	-
δ(C-H) ip + *v*($C-OCH_3$)	1535	$1536^{(SM)}$	1494	C-H bending / ring deformation
v(C-C)	1665	1666	1598	Aromatic C-C stretch / ring mode
v(C-H) sym – CH_3	2997	$3006^{(SM)}$	-	C-H stretch (on $O-CH_3$)
v(C-H) asym – CH_3	3062	$3078^{(SM)}$	2850	
v(C-H) asym – CH_3	3155	$3152^{(SM)}$	2958	

ν(C-H) asym	3202	3201	3006, 3078	aromatic C-H stretch
Guaiacol				
δ(C-H) oop + δ(O-H) oop	447	461, 488	-	-
δ(C-H) oop	751	755	742	out-of-plane C-H bend
δ(C-H) ip + ν(C-OCH_3)	1075	1074	1038	C-O stretch / in-plane C-H bend
δ(C-H) ip + δ(O-H)	1142	1141	-	-
	1234	1226	-	-
δ(C-H) ip + ν(C-OCH_3) + ν(C-OH)	1263	1265	1110	C-O stretch / in-plane C-H bend
δ(C-H) ip + ν(C-OCH_3) + ν(C-OH)	1304	1310	1222, 1262	aromatic C-O stretch
ν(C-C) + δ(O-H)	1413	1411	1366	in-plane O-H in-plane bend
γ(CH_3) + δ(C-H) ip	1508	1510	-	-
δ(C-H) ip + ν(C-OCH_3) + ν(C-OH)	1550	1557	-	-
ν(C-C)	1655	1654	1506, 1602	Aromatic C-C stretch / ring mode
ν(C-H) sym – CH_3	3011	3020	2958	C-H stretch (CH3 methyl)
ν(C-H) asym – CH_3	3079	3097	-	-
ν(C-H) asym – CH_3	3159	3197	3010	aromatic C-H stretch
ν(C-H) asym – CH_3	3209	3207	3066	aromatic C-H stretch
free ν(O-H)	3812	3773	3590	O-H stretch

ν---stretching, δ---bending, γ---scissoring, sym---symmetric, asym—asymmetric, oop---out-of-plane, in---in-plane, SM---single molecule vibrational modes. Vibrational mode description is obtained using Avogadro2 software.

References used for the experimental IR assignments:

[1] Socrates, G. *Infrared and Raman Characteristic Group Frequencies: Tables and Charts*; 3rd ed.; Wiley: Chichester, UK, 2001.

[2] Shimanouchi, T. *Tables of Molecular Vibrational Frequencies*, Consolidated Volume I; National Bureau of Standards: Washington, DC, USA, 1972; pp. 1–160.

[3] Balfour, W.J. The vibrational spectrum of anisole. *Spectrochim. Acta A* 1983, *39*, 1155–1162.

[4] Scoullos, E.V.; et al. Guaiacol Adsorption and Decomposition on Platinum: Insights from IR Spectroscopy and DFT Calculations. *J. Phys. Chem. C* 2018, *122*, 24576–24588.

Table S3 Calculated vibrational frequency (cm^{-1}) of 3,3′-dimethoxy-2,2′-biphenyldiol for six stable conformations.

Mode	ct-ct	ct-at	ct-ag	tt-tt	tt-tg	tg-tg
free ν(O-H)	3813	3784	3782	3783	3777^{SM}, 3764^{SM}	3771
bound ν(O-H)	-	3688	3659	-	-	-
ν(C-H) asym – CH_3	3157	3152^{SM}, 3162^{SM}	3146^{SM}, 3163^{SM}	3157	3158^{SM}, 3153^{SM}	3153
ν(C-H) asym – CH_3	3077	3085^{SM}	3086^{SM}, 3088^{SM}	-	3092^{SM}	3094
ν(C-H) asym – CH_3	-	3054^{SM}	-	3061^{DEG}	3062^{SM}	-
ν(C-H) sym – CH_3	3009	3015^{SM}	3015^{SM}	-	3012^{SM}	3013
ν(C-H) sym – CH_3	-	2991^{SM}	3008^{SM}	2996	2996^{SM}	-
ν(C-C)	1676	-	-		-	1640
ν(C-C)	1648	1645	1647	-	-	-

v(C-C) + δ(C-H) ip	-	1634	-	1640	1635	-
v(C-C) + *v*(C-OH) + δ(C-H) ip	1547	1535	1536	-	-	-
v(C-OCH$_3$) + *v*(C-OH) + δ(C-H) ip	1532	-	1525	1538	1533	-
v(C-C) + δ(C-H) ip	-	-	-	1526	1525	-
v(C-C) + *v*(C-OH) + δ(C-H) ip	-	-	-	-	-	1516
v(C-OH) + δ(C-H) ip	-	-	-	1516	1514	-
v(C-OCH$_3$) + *v*(C-OH) + δ(C-H) ip	-	-	-	-	-	1514
δ(CCC) ip + γ(CH$_3$) + ρ(C-H) ip	1509	-	-	-	-	-
γ(CH$_3$) + δ(C-H) ip	-	1504	1504	1502	1491	1490
γ(CH$_3$)	-	-	-	-	-	1477
δ(CH$_3$) + δ(O-H) + δ(C-H) ip	1462	1462^{SM}, 1455^{SM}	1462^{SM}	-	-	-
v(C-C) + δ(CH$_3$) + δ(O-H)	1412	1413^{SM}, 1412^{SM}	1413^{SM}	-	-	-
v(C-C) + δ(O-H)	-	-	1395^{SM}	1405	1407^{SM}	-
v(C-C) + δ(O-H)	-	-	-	-	1388^{SM}	1385
v(C-C) link + δ(C-H) ip + δ(O-H) + *v*(C-OCH$_3$)	1345	1328	-	1354	1352	1349
v(C-C) + ρ(C-H) ip + δ(O-H)	1317	-	1325	-	-	1315
ρ(C-H) ip + *v*(C-OH)	1308	-	-	-	-	-
v(C-C) link + *v*(C-OH) + ρ(C-H) ip	-	-	1303	-	-	-
v(C-OCH$_3$) + *v*(C-OH) + δ(C-H) ip	1297	-	1298	-	1292^{SM}, 1277^{SM}	-
v(C-OCH$_3$) + δ(O-H) + δ(C-H) ip	-	1299	1278	1287, 1275	-	1283
v(C-OH) + δ(C-H) ip + δ(O-H)	-	1273			-	-
v(C-OH) + δ(C-H) ip	-	-	-	-	1267	
v(C-OCH$_3$) + *v*(C-OH) + δ(C-H) ip	1266	1252^{SM}	1251^{SM}	-		-
δ(O-CH$_3$) + δ(O-H) + ρ(C-H) ip	1214	1223	1218	-	-	1203
δ(O-H) + δ(C-H) ip	1169	1174	1166	1162	1155	-
γ(C-H) ip	1106	1121^{SM}, 1109^{SM}	-	1114	1099^{SM}, 1112^{SM}	1097
v(O-CH$_3$) + δ(C-H) ip	-	-	1103	-	-	-
v(O-CH$_3$) + δ(CCC) ip	1076	1075	1054	1072	1051	1045
δ(C-H) ip + δ(CCC) ip	928	929	937	-	-	-
δ(CCC) ip	-	-	-	854	861	867
ω(C-H) oop + δ(CCC) oop	-	-	-	-	-	812
ω(C-H) oop + δ(CCC) oop	777	780	782	788	-	-
ω(C-H) oop + δ(CCC) oop	745	745	749^{SM}	750	756^{SM}, 762^{SM}	761
δ(O-H) + δ(CCC) ip	-	549	574	-	-	-
δ(O-H)	-	-	536^{SM}	-	-	509
δ(O-H)	-	-	-	-	497	486
δ(O-H)	439	-	-	423	-	-

v---stretching, δ---bending, γ---scissoring, ω---wagging, τ---twisting, torsion, ρ---rocking, link---linkage, sym---symmetric, asym—asymmetric, oop---out-of-plane, ip---in-plane, SM---single molecule vibrational modes. Vibrational mode description is obtained using Avogadro2 software.

Table S4 Comparation of relative electronic energy of benzene and guaiacol dimers obtained by APFD (M1), M06-2X (M2) and ωB97X-D (M3).

No.	Molecules	El.En.Rel (eV)		
		M1	M2	M3
1.	ParO benzene dimer	0.00	0.00	0.00
2.	PerT benzene dimer	0.02	0.02	0.01
3.	ParO1 guaiacol dimer	0.00	0.00	0.00
4.	ParO2 guaiacol dimer	0.05	0.05	0.06
5.	ParO3 guaiacol dimer	0.10	0.09	0.12
6.	ParO4 guaiacol dimer	0.13	0.19	0.14
7.	ParO5 guaiacol dimer	0.15	0.13	0.15
8.	ParO6 guaiacol dimer	0.15	0.16	0.00
